# Crystal structures of two bis­(iodo­meth­yl)benzene derivatives: similarities and differences in the crystal packing

**DOI:** 10.1107/S2056989015021295

**Published:** 2015-11-18

**Authors:** C. John McAdam, Lyall R. Hanton, Stephen C. Moratti, Jim Simpson

**Affiliations:** a Department of Chemistry, University of Otago, PO Box 56, Dunedin, New Zealand

**Keywords:** crystal structure, bis­(iodo­meth­yl)benzene derivatives, C—H⋯I hydrogen bonds, C—H⋯π(ring) contacts, π–π contacts, I⋯I halogen bonds

## Abstract

The isomeric derivatives 1,2-bis­(iodo­meth­yl)benzene, (I), and 1,3-bis­(iodo­meth­yl)benzene (II), both C_8_H_8_I_2_, were prepared by metathesis from their di­bromo analogues. The *ortho*-derivative, (I), lies about a crystallographic twofold axis that bis­ects the C—C bond between the two iodo­methyl substituents. The packing in (I) relies solely on C—H⋯I hydrogen bonds supported by weak parallel slipped π–π stacking inter­actions [inter-centroid distance = 4.0569 (11) Å, inter-planar distance = 3.3789 (8) Å and slippage = 2.245 Å]. While C—H⋯I hydrogen bonds are also found in the packing of (II), type II, I⋯I halogen bonds [I⋯I = 3.8662 (2) Å] and C—H⋯π contacts feature prominently in stabilizing the three-dimensional structure.

## Abstract 2

The mol­ecular and crystal structures of two isomeric bis­(iodo­meth­yl)benzene derivatives are reported. A comparison is made of the inter­molecular contacts stabilizing the packing in the two closely related systems.

## Chemical context   

The isomeric xylene derivatives reported here, 1,2-bis­(iodo­meth­yl)benzene, (I), and 1,3-bis­(iodo­meth­yl)benzene (II), are useful synthons for the preparation of a range of organic compounds. (I) is used particularly in the synthesis of polycyclic aromatic systems (see for example: Takahashi *et al.* 2006 ▸; Abreu *et al.*, 2010 ▸; Wang *et al.*, 2012 ▸). Similarly (II) has been used in polymer formation (Pandya & Gibson, 1991 ▸), in the synthesis of meta­cyclo­phanes (Ramming & Gleiter, 1997 ▸) and to provide aromatic spacers in organic synthesis (Kida *et al.*, 2005 ▸). Our inter­est in such compounds is as components of ionene polymers. The compounds were readily prepared by metathesis from the bis­(bromo­meth­yl)benzene derivatives.
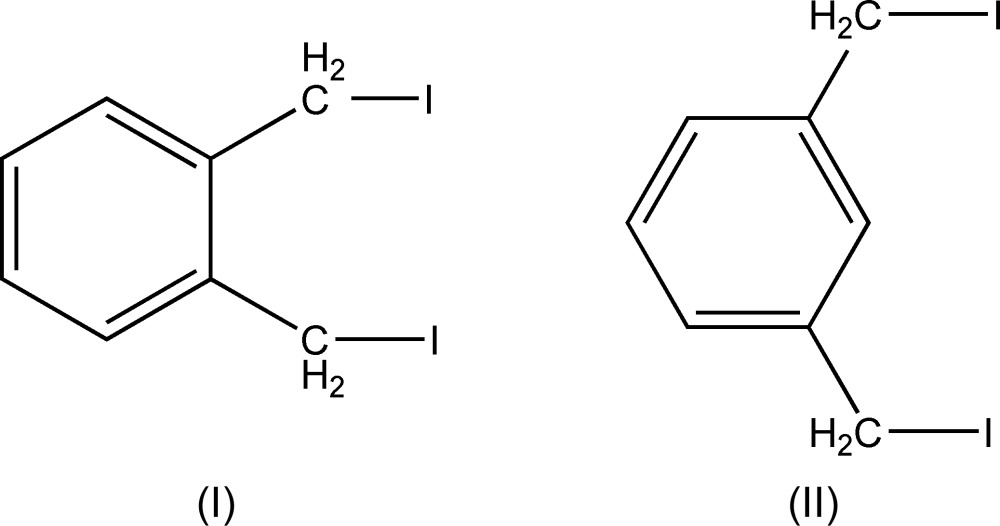


## Structural commentary   

**Figure 1:**
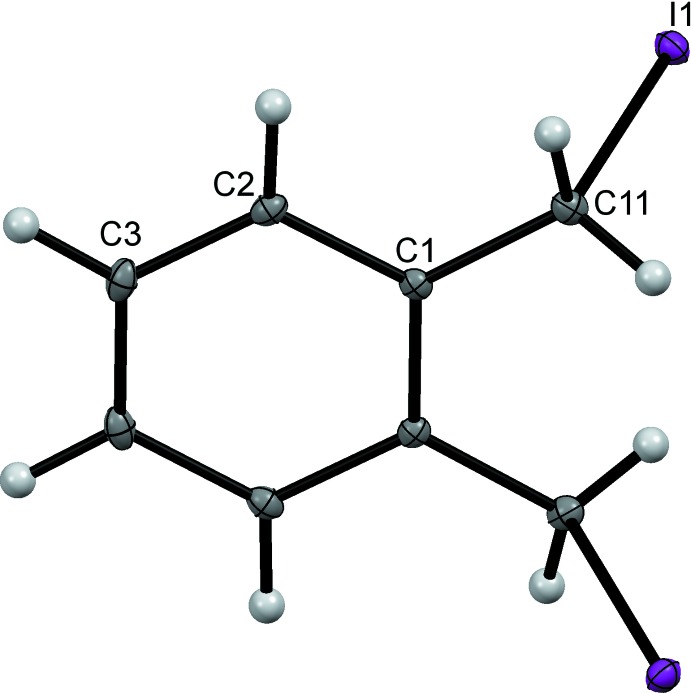
The mol­ecular structure of compound (I), with displacement ellipsoids drawn at the 50% probability level. The unlabelled atoms are related to labelled atoms by the symmetry operation (−*x* + 1, *y*, −*z* + 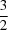).

The mol­ecular structures of 1,2-bis­(iodo­meth­yl)benzene, (I), and 1,3-bis­(iodo­meth­yl)benzene, (II), are shown in Figs. 1 ▸ and 2 ▸ and are sufficiently similar to be discussed together. Each comprises a benzene ring with two iodo­methyl substituents in the 1,2- and 1,3-positions for (I) and (II) respectively. The mol­ecule of (I) lies about a twofold axis that bis­ects the C—C bond between the two iodo­methyl substituents. For each mol­ecule the C—I bonds of the substituents point away from opposite faces of the benzene rings with the C—C—I planes almost orthogonal to the ring planes; dihedral angles = 87.99 (14)° for (I) and 82.23 (14) and 83.61 (15)° for (II). The C1—C11 and C11—I1 bond lengths in (I) and C1—C11, C11—I1, C3—C31 and C31—I3 in (II) are reasonably self-consistent and also compare well with those found in the isomeric 1,4-bis­(iodo­meth­yl)benzene (McAdam *et al.* 2009 ▸).

## Supra­molecular features   

### Crystal packing for (I)   

**Figure 3:**
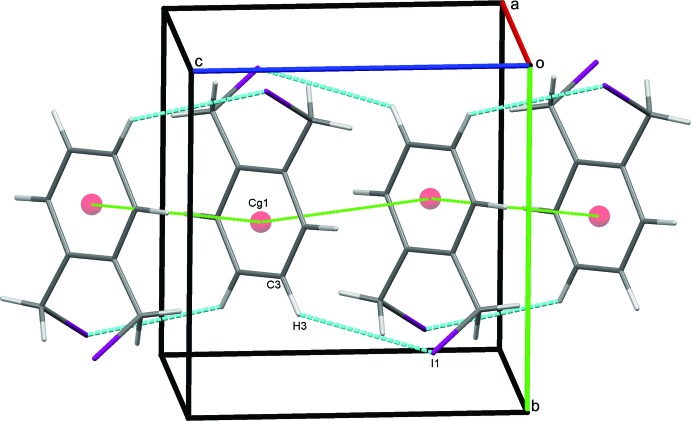
π–π stacking inter­actions (green dotted lines) supported by C—H⋯I hydrogen bonds for (I). Hydrogen bonds in this and subsequent figures are drawn as blue dashed lines.

**Figure 4:**
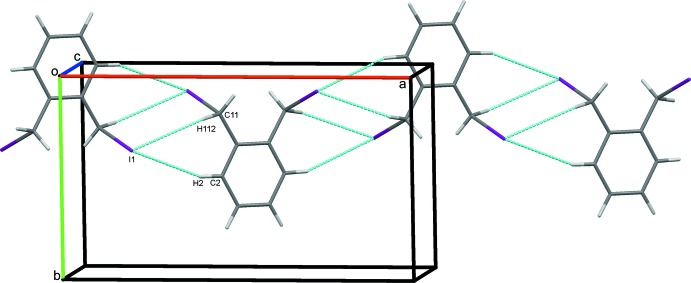
Chains of mol­ecules of (I) in [101].

**Figure 5:**
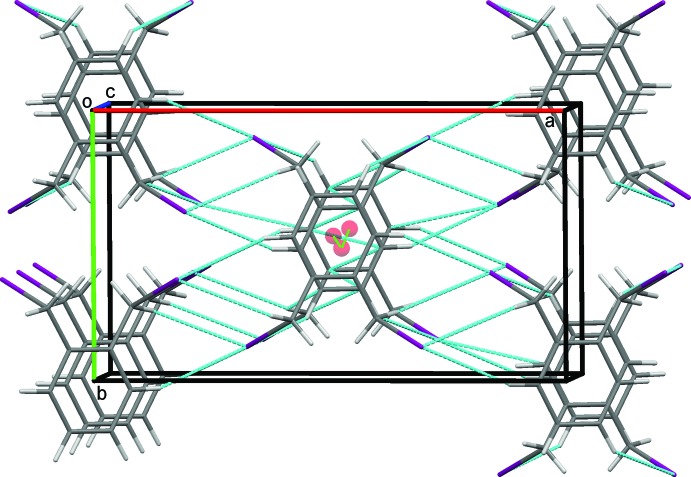
Overall packing for (I) viewed along the *c*-axis direction.

In the crystal of (I), weak parallel slipped π–π stacking inter­actions [inter-centroid distance = 4.0569 (11) Å, inter-planar distance = 3.3789 (8) Å, slippage = 2.245 Å], between the benzene rings of inversion-related mol­ecules are supported by C3—H3⋯I1 hydrogen bonds, Table 1 ▸, to link mol­ecules in a head-to tail-fashion, stacking them along *c*, Fig. 3 ▸. In addition, the iodine atoms act as bifurcated acceptors, forming weak C2—H2⋯I1 and C11—H112⋯I1 hydrogen bonds generating 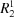
(6) ring motifs (Bernstein *et al.*, 1995 ▸). These contacts link the mol­ecules into zigzag chains along [101], Fig. 4 ▸. These contacts combine to link stacked columns of mol­ecules through weak C—H⋯I hydrogen bonds and generate a three dimensional network structure, Fig. 5 ▸.

### Crystal packing for (II)   

**Figure 6:**
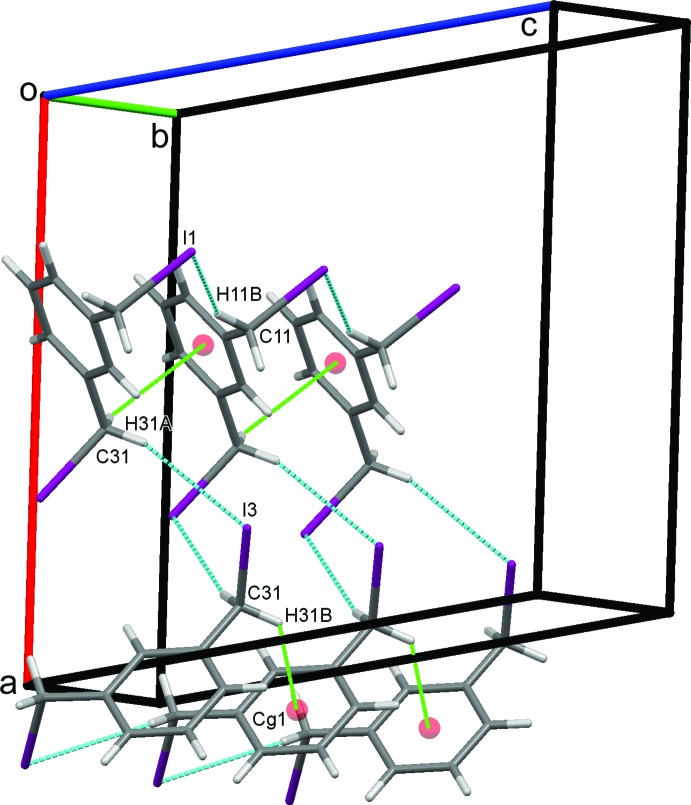
Double chains of mol­ecules of (II) formed by a series of C31—H31*B*⋯*Cg*1 contacts (green dotted lines) linked by C—H⋯I hydrogen bonds.

**Figure 7:**
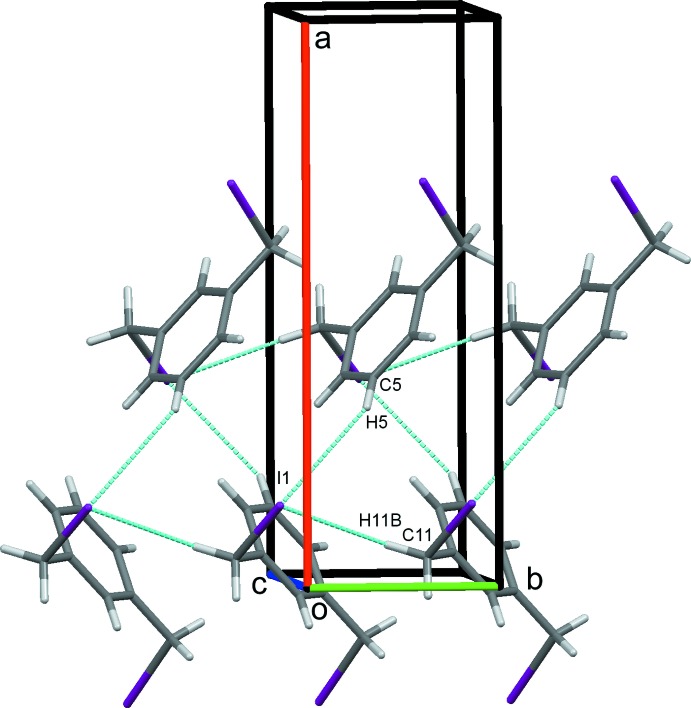
Sheets of mol­ecules of (II) in the *ab* plane formed by C—H⋯I. hydrogen bonds.

**Figure 8:**
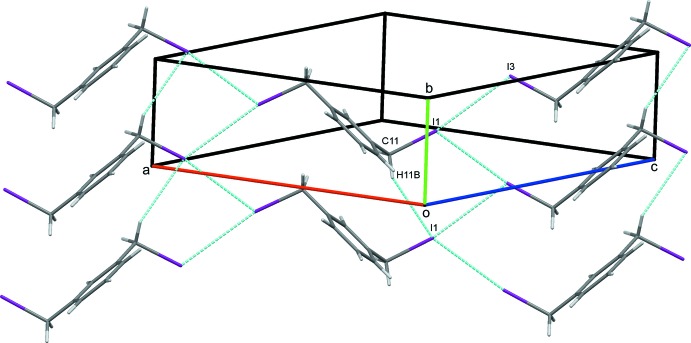
Sheets of mol­ecules of (II) in the (101) plane formed by I⋯I halogen bonds, blue dashed lines, supported by C—H⋯I hydrogen bonds.

**Figure 9:**
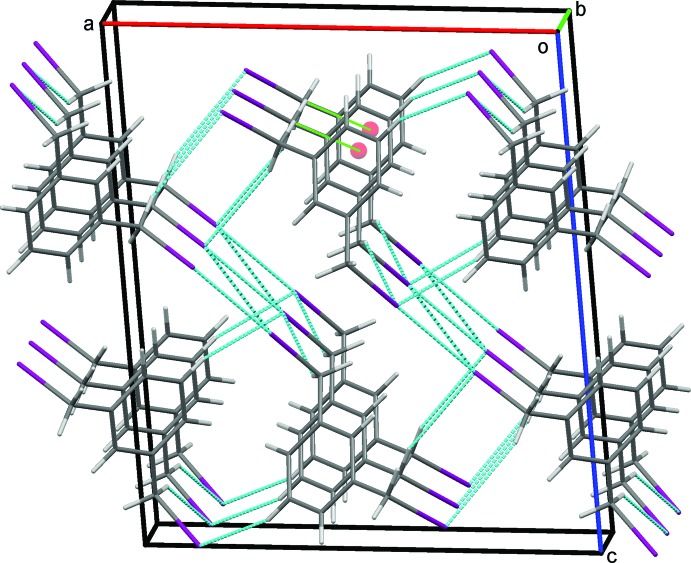
Overall packing for (II) viewed along the *b*-axis direction.

In the crystal of (II), C11—H11*B*⋯I1 hydrogen bonds, Table 2 ▸, form a column supported by a series of C31—H31*B*⋯*Cg*1 contacts. C31—H31*A*⋯I3 hydrogen bonds link these in an obverse fashion, forming double chains along *b*, Fig. 6 ▸. C5—H5⋯I1 hydrogen bonds, Fig. 7 ▸, link the double chains into sheets in the *ab* plane. An extensive series of I1⋯I3 halogen bonds Fig. 8 ▸, I1⋯I3^v,vi^ = 3.8662 (2) Å; symmetry codes: (v) = −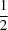 + *x*, 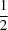 − *y*, 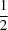 + *z;* (vi) = 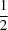 + *x*, 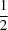 − *y*, −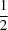 + *z* (Desiraju *et al.*, 2013 ▸; Metrangolo *et al.*, 2008 ▸), extend the structure in the third dimension, Fig. 9 ▸. The angles C11—I1—I3 = 117° and C31—I3—I1 = 165° characterize this halogen bond as type II (Pedireddi *et al.*, 1994 ▸).

## Database survey   

A search of the Cambridge Structural Database (Version 5.36 with three updates; Groom & Allen, 2014 ▸) for mol­ecules incorporating a C_6_CH_2_I fragment surprisingly generated only five hits for iodo­methyl­benzene derivatives. One of these is the isomeric 1,4-bis­(iodo­meth­yl)benzene reported by us previously (McAdam *et al.*, 2009 ▸), while two others are the organic compounds 2-(iodo­meth­yl)-1,3,5-tri­methyl­benzene (Bats, 2014 ▸) and 3′-iodo-5′-(iodo­meth­yl)biphenyl-4-carbo­nitrile (He *et al.*, 2013 ▸). The other two entries are metal complexes (Martínez-García *et al.*, 2010 ▸; Rivada-Wheelaghan *et al.*, 2012 ▸). In one of these, the iodine atom of the iodo­methyl unit was found to act as a ligand to a platinum(II) nucleus (Rivada-Wheelaghan *et al.*, 2012 ▸). The structures of both the chloro- and bromo-analogues of 1,2-bis­(iodo­meth­yl)benzene (Basaran *et al.*, 1992 ▸; Jones & Kus, 2007 ▸) and 1,3-bis­(iodo­meth­yl)benzene (Sanders *et al.*, 2013 ▸; Li *et al.*, 2006 ▸; Jones & Kus, 2007 ▸) have also been reported. Inter­estingly, 1,3-bis(bromo­meth­yl)benzene is isostructural with (II) and the packing features for the two compounds are identical, apart from somewhat increased distances for the iodo compound. For example I1⋯I3 = 3.8662 (2) Å for (II) but the equivalent Br⋯Br distance is 3.6742 (3) Å for the *meta*-di­bromo analogue (Jones & Kus, 2007 ▸). Similar isostructural behaviour is observed for *para*-bis­(iodo­meth­yl)benzene (McAdam *et al.*, 2009 ▸) and its di­bromo analogue (Jones & Kus, 2007 ▸). However, in contrast, despite (I) and the *ortho*-di­bromo analogue both displaying twofold symmetry, compound (I) crystallizes in the monoclinic space group *C*2/*c* while that for the di­bromo counterpart is found to be ortho­rhom­bic, *Fdd*2 (Jones & Kus, 2007 ▸).

## Synthesis and crystallization   

Preparation of the title compounds was based on literature methods (Moore & Stupp, 1986 ▸; Kida *et al.*, 2005 ▸). The appropriate bis­(bromo­meth­yl)benzene (1.32 g, 5 mmol) was refluxed for 7 h with sodium iodide (2.25 g, 15 mmol) in acetone (25 ml). The solution was allowed to cool overnight, the crystals that developed were rinsed gently with water to remove sodium bromide and air dried. The product was recrystallized a second time from acetone to give X-ray quality crystals. Confirmation of the metathesised (iodo) product was by microanalysis and mass spectroscopy. ^13^C NMR spectra of the di­iodo compounds are distinct from those of their di­bromo precursors.

Compound (I): Analysis calculated for C_8_H_8_I_2_: C, 26.84; H, 2.25%. Found: C, 26.86; H, 2.14%. ^13^C NMR (δ p.p.m.): 137.4, 130.8, 129.0, 1.8.

Compound (II): Analysis calculated for C_8_H_8_I_2_: C, 26.84; H, 2.25%. Found: C, 26.63; H, 2.19%. ^13^C NMR (δ p.p.m.): 140.0, 129.4, 129.0, 128.4, 4.9.

## Refinement   

Crystal data, data collection and structure refinement details are summarized in Table 3 ▸. All H atoms were refined using a riding model with *d*(C—H) = 0.95 Å, *U*_iso_ = 1.2*U*_eq_(C) for aromatic and 0.99 Å, *U*_iso_ = 1.2*U*_eq_(C) for CH_2_ H atoms. For (I), a low-angle reflection with *F*_o_ << *F*_c_, that may have been affected by the beam-stop, was omitted from the final refinement cycles.

## Supplementary Material

Crystal structure: contains datablock(s) global, I, II. DOI: 10.1107/S2056989015021295/su5235sup1.cif

Structure factors: contains datablock(s) I. DOI: 10.1107/S2056989015021295/su5235Isup2.hkl

Structure factors: contains datablock(s) II. DOI: 10.1107/S2056989015021295/su5235IIsup3.hkl

Supporting information file. DOI: 10.1107/S2056989015021295/su5235Isup4.cml

Supporting information file. DOI: 10.1107/S2056989015021295/su5235IIsup5.cml

CCDC references: 1436014, 1436013

Additional supporting information:  crystallographic information; 3D view; checkCIF report

## Figures and Tables

**Figure 2:**
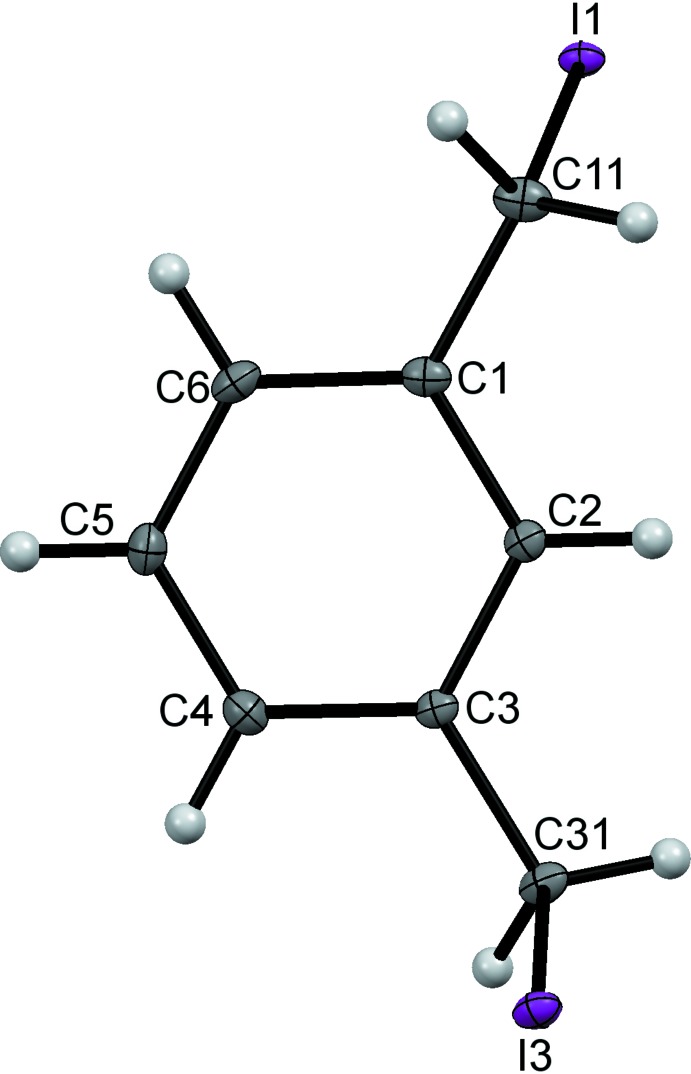
The mol­ecular structure of compound (II), with displacement ellipsoids drawn at the 50% probability level.

**Table 1:** Hydrogen-bond geometry (Å, °) for (I)

*D*—H⋯*A*	*D*—H	H⋯*A*	*D*⋯*A*	*D*—H⋯*A*
C3—H3⋯I1^i^	0.95	3.38	4.046 (2)	129
C11—H112⋯I1^ii^	0.99	3.33	4.179 (2)	145
C2—H2⋯I1^ii^	0.95	3.36	4.257 (2)	158

Symmetry codes: (i) 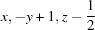; (ii) 
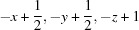
.

**Table 2:** Hydrogen-bond geometry (Å, °) for (II) *Cg* is the centroid of the C1–C6 ring.

*D*—H⋯*A*	*D*—H	H⋯*A*	*D*⋯*A*	*D*—H⋯*A*
C11—H11*B*⋯I1^i^	0.99	3.22	4.060 (3)	144
C5—H5⋯I1^ii^	0.95	3.25	4.078 (3)	147
C31—H31*A*⋯I3^iii^	0.99	3.27	4.224 (3)	162
C31—H31*A*⋯*Cg* ^iv^	0.99	2.84	3.453 (3)	121

Symmetry codes: (i) 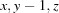; (ii) 
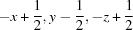
; (iii) 
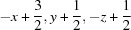
; (iv) 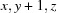.

**Table 3:** Experimental details

	(I)	(II)
Crystal data		
Chemical formula	C_8_H_8_I_2_	C_8_H_8_I_2_
*M*_r_	357.94	357.94
Crystal system, space group	Monoclinic, *C*2/*c*	Monoclinic, *P*2_1_/*n*
Temperature (K)	90	90
*a*, *b*, *c* (Å)	14.5485 (5), 8.0461 (3), 8.0582 (3)	13.5323 (3), 4.5464 (1), 15.6269 (4)
β (°)	101.637 (2)	95.203 (1)
*V* (Å^3^)	923.89 (6)	957.46 (4)
*Z*	4	4
Radiation type	Mo *K*α	Mo *K*α
μ (mm^−1^)	6.74	6.50
Crystal size (mm)	0.31 × 0.17 × 0.15	0.45 × 0.06 × 0.05

Data collection		
Diffractometer	Bruker APEXII CCD area detector	Bruker APEXII CCD area detector
Absorption correction	Multi-scan (*SADABS*; Bruker, 2013 ▸)	Multi-scan (*SADABS*; Bruker, 2013 ▸)
*T*_min_, *T*_max_	0.534, 1.000	0.569, 1.000
No. of measured, independent and observed [*I* > 2σ(*I*)] reflections	8422, 1667, 1552	16804, 3435, 2826
*R*_int_	0.030	0.033
(sin θ/λ)_max_ (Å^−1^)	0.775	0.775

Refinement		
*R*[*F* ^2^ > 2σ(*F* ^2^)], *wR*(*F* ^2^), *S*	0.018, 0.044, 1.15	0.024, 0.048, 1.06
No. of reflections	1667	3435
No. of parameters	46	91
H-atom treatment	H-atom parameters constrained	H-atom parameters constrained
Δρ_max_, Δρ_min_ (e Å^−3^)	0.52, −1.23	1.24, −0.77

Computer programs: *APEX2* and *SAINT* (Bruker, 2013 ▸), *SHELXT* (Sheldrick, 2015*a* ▸), *SHELXL2014* (Sheldrick, 2015*b* ▸), *TITAN2000* (Hunter & Simpson, 1999 ▸), *Mercury* (Macrae *et al.*, 2008 ▸), *enCIFer* (Allen *et al.*, 2004 ▸), *PLATON* (Spek, 2009 ▸), *WinGX* (Farrugia, 2012 ▸) and *publCIF* (Westrip, 2010 ▸).

## supplementary crystallographic information

### (I) 1,2-Bis(iodomethyl)benzene Crystal data

**Table:**

C_8_H_8_I_2_	*F*(000) = 648
*M**_r_* = 357.94	*D*_x_ = 2.573 Mg m^−^^3^
Monoclinic, *C*2/*c*	Mo *K*α radiation, λ = 0.71073 Å
*a* = 14.5485 (5) Å	Cell parameters from 5091 reflections
*b* = 8.0461 (3) Å	θ = 2.6–32.9°
*c* = 8.0582 (3) Å	µ = 6.74 mm^−^^1^
β = 101.637 (2)°	*T* = 90 K
*V* = 923.89 (6) Å^3^	Block, colourless
*Z* = 4	0.31 × 0.17 × 0.15 mm

### (I) 1,2-Bis(iodomethyl)benzene Data collection

**Table:**

Bruker APEXII CCD area-detector diffractometer	1552 reflections with *I* > 2σ(*I*)
Radiation source: fine-focus sealed tube	*R*_int_ = 0.030
ω scans	θ_max_ = 33.4°, θ_min_ = 2.9°
Absorption correction: multi-scan (SADABS; Bruker, 2013)	*h* = −21→21
*T*_min_ = 0.534, *T*_max_ = 1.000	*k* = −11→12
8422 measured reflections	*l* = −12→10
1667 independent reflections	

### (I) 1,2-Bis(iodomethyl)benzene Refinement

**Table:**

Refinement on *F*^2^	0 restraints
Least-squares matrix: full	Hydrogen site location: inferred from neighbouring sites
*R*[*F*^2^ > 2σ(*F*^2^)] = 0.018	H-atom parameters constrained
*wR*(*F*^2^) = 0.044	*w* = 1/[σ^2^(*F*_o_^2^) + (0.0175*P*)^2^ + 1.2212*P*] where *P* = (*F*_o_^2^ + 2*F*_c_^2^)/3
*S* = 1.15	(Δ/σ)_max_ = 0.002
1667 reflections	Δρ_max_ = 0.52 e Å^−^^3^
46 parameters	Δρ_min_ = −1.23 e Å^−^^3^

### (I) 1,2-Bis(iodomethyl)benzene Special details

**Table:**

Geometry. All e.s.d.'s (except the e.s.d. in the dihedral angle between two l.s. planes) are estimated using the full covariance matrix. The cell e.s.d.'s are taken into account individually in the estimation of e.s.d.'s in distances, angles and torsion angles; correlations between e.s.d.'s in cell parameters are only used when they are defined by crystal symmetry. An approximate (isotropic) treatment of cell e.s.d.'s is used for estimating e.s.d.'s involving l.s. planes.
Refinement. One low angle reflection with *F_o_* << Fc was omitted from the final refinement cycles.

### (I) 1,2-Bis(iodomethyl)benzene Fractional atomic coordinates and isotropic or equivalent isotropic displacement parameters (Å^2^)

**Table:**

	*x*	*y*	*z*	*U*_iso_*/*U*_eq_	
I1	0.31503 (2)	0.11885 (2)	0.75250 (2)	0.01529 (5)	
C11	0.41526 (13)	0.2215 (2)	0.6102 (3)	0.0142 (3)	
H111	0.4651	0.1386	0.6070	0.017*	
H112	0.3826	0.2433	0.4921	0.017*	
C1	0.45886 (13)	0.3782 (2)	0.6864 (2)	0.0111 (3)	
C2	0.41839 (13)	0.5301 (2)	0.6268 (3)	0.0136 (3)	
H2	0.3623	0.5307	0.5427	0.016*	
C3	0.45882 (14)	0.6802 (2)	0.6886 (3)	0.0156 (4)	
H3	0.4304	0.7823	0.6470	0.019*	

### (I) 1,2-Bis(iodomethyl)benzene Atomic displacement parameters (Å^2^)

**Table:**

	*U*^11^	*U*^22^	*U*^33^	*U*^12^	*U*^13^	*U*^23^
I1	0.01370 (7)	0.01474 (7)	0.01747 (8)	−0.00335 (4)	0.00325 (5)	0.00078 (4)
C11	0.0139 (8)	0.0163 (8)	0.0132 (9)	−0.0016 (6)	0.0044 (7)	−0.0027 (7)
C1	0.0115 (8)	0.0123 (8)	0.0102 (8)	−0.0008 (5)	0.0039 (6)	−0.0001 (6)
C2	0.0131 (8)	0.0158 (8)	0.0122 (9)	0.0026 (6)	0.0035 (7)	0.0011 (7)
C3	0.0212 (9)	0.0122 (8)	0.0153 (9)	0.0026 (7)	0.0085 (7)	0.0031 (7)

### (I) 1,2-Bis(iodomethyl)benzene Geometric parameters (Å, º)

**Table:**

I1—C11	2.1902 (19)	C1—C1^i^	1.410 (4)
C11—C1	1.487 (3)	C2—C3	1.391 (3)
C11—H111	0.9900	C2—H2	0.9500
C11—H112	0.9900	C3—C3^i^	1.392 (4)
C1—C2	1.399 (3)	C3—H3	0.9500
			
C1—C11—I1	112.15 (13)	C1^i^—C1—C11	121.93 (11)
C1—C11—H111	109.2	C3—C2—C1	121.16 (18)
I1—C11—H111	109.2	C3—C2—H2	119.4
C1—C11—H112	109.2	C1—C2—H2	119.4
I1—C11—H112	109.2	C2—C3—C3^i^	119.72 (12)
H111—C11—H112	107.9	C2—C3—H3	120.1
C2—C1—C1^i^	119.10 (11)	C3^i^—C3—H3	120.1
C2—C1—C11	118.94 (18)		
			
I1—C11—C1—C2	−93.41 (19)	C11—C1—C2—C3	−177.12 (17)
I1—C11—C1—C1^i^	88.3 (2)	C1—C2—C3—C3^i^	0.2 (3)
C1^i^—C1—C2—C3	1.2 (3)		

Symmetry code: (i) −*x*+1, *y*, −*z*+3/2.

### (I) 1,2-Bis(iodomethyl)benzene Hydrogen-bond geometry (Å, º)

**Table:**

*D*—H···*A*	*D*—H	H···*A*	*D*···*A*	*D*—H···*A*
C3—H3···I1^ii^	0.95	3.38	4.046 (2)	129
C11—H112···I1^iii^	0.99	3.33	4.179 (2)	145
C2—H2···I1^iii^	0.95	3.36	4.257 (2)	158

Symmetry codes: (ii) *x*, −*y*+1, *z*−1/2; (iii) −*x*+1/2, −*y*+1/2, −*z*+1.

### (II) 1,3-Bis(iodomethyl)benzene Crystal data

**Table:**

C_8_H_8_I_2_	*F*(000) = 648
*M**_r_* = 357.94	*D*_x_ = 2.483 Mg m^−^^3^
Monoclinic, *P*2_1_/*n*	Mo *K*α radiation, λ = 0.71073 Å
*a* = 13.5323 (3) Å	θ = 2.6–33.0°
*b* = 4.5464 (1) Å	µ = 6.50 mm^−^^1^
*c* = 15.6269 (4) Å	*T* = 90 K
β = 95.203 (1)°	Needle, colourless
*V* = 957.46 (4) Å^3^	0.45 × 0.06 × 0.05 mm
*Z* = 4	

### (II) 1,3-Bis(iodomethyl)benzene Data collection

**Table:**

Bruker APEXII CCD area-detector diffractometer	2826 reflections with *I* > 2σ(*I*)
Radiation source: fine-focus sealed tube	*R*_int_ = 0.033
ω scans	θ_max_ = 33.4°, θ_min_ = 3.0°
Absorption correction: multi-scan (SADABS; Bruker, 2013)	*h* = −20→20
*T*_min_ = 0.569, *T*_max_ = 1.000	*k* = −6→5
16804 measured reflections	*l* = −23→24
3435 independent reflections	

### (II) 1,3-Bis(iodomethyl)benzene Refinement

**Table:**

Refinement on *F*^2^	0 restraints
Least-squares matrix: full	Hydrogen site location: inferred from neighbouring sites
*R*[*F*^2^ > 2σ(*F*^2^)] = 0.024	H-atom parameters constrained
*wR*(*F*^2^) = 0.048	*w* = 1/[σ^2^(*F*_o_^2^) + (0.0109*P*)^2^ + 1.4343*P*] where *P* = (*F*_o_^2^ + 2*F*_c_^2^)/3
*S* = 1.06	(Δ/σ)_max_ = 0.002
3435 reflections	Δρ_max_ = 1.24 e Å^−^^3^
91 parameters	Δρ_min_ = −0.77 e Å^−^^3^

### (II) 1,3-Bis(iodomethyl)benzene Special details

**Table:**

Geometry. All e.s.d.'s (except the e.s.d. in the dihedral angle between two l.s. planes) are estimated using the full covariance matrix. The cell e.s.d.'s are taken into account individually in the estimation of e.s.d.'s in distances, angles and torsion angles; correlations between e.s.d.'s in cell parameters are only used when they are defined by crystal symmetry. An approximate (isotropic) treatment of cell e.s.d.'s is used for estimating e.s.d.'s involving l.s. planes.

### (II) 1,3-Bis(iodomethyl)benzene Fractional atomic coordinates and isotropic or equivalent isotropic displacement parameters (Å^2^)

**Table:**

	*x*	*y*	*z*	*U*_iso_*/*U*_eq_	
I1	0.35561 (2)	0.36401 (4)	0.46514 (2)	0.01445 (4)	
C11	0.4498 (2)	0.1469 (6)	0.37746 (17)	0.0197 (5)	
H11A	0.5193	0.1461	0.4034	0.024*	
H11B	0.4282	−0.0599	0.3690	0.024*	
C1	0.44471 (19)	0.2984 (6)	0.29275 (16)	0.0152 (5)	
C2	0.51858 (18)	0.4993 (5)	0.27547 (16)	0.0138 (5)	
H2	0.5707	0.5419	0.3185	0.017*	
C3	0.51650 (18)	0.6379 (5)	0.19574 (16)	0.0128 (4)	
C31	0.59593 (19)	0.8514 (6)	0.17788 (17)	0.0175 (5)	
H31A	0.6261	0.9352	0.2326	0.021*	
H31B	0.5667	1.0148	0.1421	0.021*	
I3	0.71036 (2)	0.63230 (4)	0.11079 (2)	0.01692 (5)	
C4	0.43920 (19)	0.5755 (6)	0.13244 (16)	0.0171 (5)	
H4	0.4369	0.6696	0.0780	0.021*	
C5	0.36591 (19)	0.3753 (6)	0.14961 (17)	0.0179 (5)	
H5	0.3138	0.3322	0.1066	0.022*	
C6	0.36832 (19)	0.2379 (6)	0.22919 (18)	0.0178 (5)	
H6	0.3177	0.1021	0.2404	0.021*	

### (II) 1,3-Bis(iodomethyl)benzene Atomic displacement parameters (Å^2^)

**Table:**

	*U*^11^	*U*^22^	*U*^33^	*U*^12^	*U*^13^	*U*^23^
I1	0.01484 (7)	0.01475 (9)	0.01451 (8)	0.00033 (6)	0.00534 (5)	0.00083 (6)
C11	0.0223 (12)	0.0167 (13)	0.0216 (12)	0.0064 (11)	0.0091 (10)	0.0020 (11)
C1	0.0174 (11)	0.0125 (12)	0.0167 (11)	0.0032 (10)	0.0065 (9)	0.0003 (9)
C2	0.0149 (11)	0.0112 (12)	0.0155 (11)	0.0011 (9)	0.0035 (9)	−0.0025 (9)
C3	0.0139 (10)	0.0097 (11)	0.0153 (10)	0.0010 (9)	0.0044 (8)	−0.0013 (9)
C31	0.0191 (12)	0.0131 (13)	0.0215 (12)	−0.0020 (10)	0.0083 (10)	−0.0037 (10)
I3	0.01582 (8)	0.01765 (9)	0.01833 (8)	−0.00134 (6)	0.00728 (6)	−0.00027 (6)
C4	0.0184 (11)	0.0182 (13)	0.0148 (11)	0.0024 (10)	0.0016 (9)	0.0005 (10)
C5	0.0152 (11)	0.0191 (13)	0.0190 (12)	0.0004 (10)	−0.0012 (9)	−0.0044 (10)
C6	0.0153 (11)	0.0147 (13)	0.0243 (13)	−0.0029 (10)	0.0057 (10)	−0.0010 (11)

### (II) 1,3-Bis(iodomethyl)benzene Geometric parameters (Å, º)

**Table:**

I1—C11	2.189 (3)	C3—C31	1.493 (3)
I1—I3^i^	3.8662 (2)	C31—I3	2.187 (2)
C11—C1	1.488 (4)	C31—H31A	0.9900
C11—H11A	0.9900	C31—H31B	0.9900
C11—H11B	0.9900	C4—C5	1.390 (4)
C1—C6	1.394 (4)	C4—H4	0.9500
C1—C2	1.399 (3)	C5—C6	1.390 (4)
C2—C3	1.394 (3)	C5—H5	0.9500
C2—H2	0.9500	C6—H6	0.9500
C3—C4	1.402 (3)		
			
C11—I1—I3^i^	117.47 (7)	C3—C31—I3	110.27 (16)
C1—C11—I1	111.45 (17)	C3—C31—H31A	109.6
C1—C11—H11A	109.3	I3—C31—H31A	109.6
I1—C11—H11A	109.3	C3—C31—H31B	109.6
C1—C11—H11B	109.3	I3—C31—H31B	109.6
I1—C11—H11B	109.3	H31A—C31—H31B	108.1
H11A—C11—H11B	108.0	C5—C4—C3	119.7 (2)
C6—C1—C2	119.2 (2)	C5—C4—H4	120.1
C6—C1—C11	120.9 (2)	C3—C4—H4	120.1
C2—C1—C11	119.9 (2)	C6—C5—C4	120.5 (2)
C3—C2—C1	120.7 (2)	C6—C5—H5	119.7
C3—C2—H2	119.6	C4—C5—H5	119.7
C1—C2—H2	119.6	C5—C6—C1	120.3 (2)
C2—C3—C4	119.5 (2)	C5—C6—H6	119.9
C2—C3—C31	120.3 (2)	C1—C6—H6	119.9
C4—C3—C31	120.2 (2)		
			
I1—C11—C1—C6	−83.6 (3)	C4—C3—C31—I3	−83.7 (3)
I1—C11—C1—C2	97.9 (2)	C2—C3—C4—C5	−0.3 (4)
C6—C1—C2—C3	−0.1 (4)	C31—C3—C4—C5	179.7 (2)
C11—C1—C2—C3	178.4 (2)	C3—C4—C5—C6	0.4 (4)
C1—C2—C3—C4	0.2 (4)	C4—C5—C6—C1	−0.3 (4)
C1—C2—C3—C31	−179.8 (2)	C2—C1—C6—C5	0.2 (4)
C2—C3—C31—I3	96.4 (2)	C11—C1—C6—C5	−178.4 (2)

Symmetry code: (i) *x*−1/2, −*y*+1/2, *z*+1/2.

### (II) 1,3-Bis(iodomethyl)benzene Hydrogen-bond geometry (Å, º)

*Cg* is the centroid of the C1–C6 ring.

**Table:**

*D*—H···*A*	*D*—H	H···*A*	*D*···*A*	*D*—H···*A*
C11—H11*B*···I1^ii^	0.99	3.22	4.060 (3)	144
C5—H5···I1^iii^	0.95	3.25	4.078 (3)	147
C31—H31*A*···I3^iv^	0.99	3.27	4.224 (3)	162
C31—H31*A*···*Cg*^v^	0.99	2.84	3.453 (3)	121

Symmetry codes: (ii) *x*, *y*−1, *z*; (iii) −*x*+1/2, *y*−1/2, −*z*+1/2; (iv) −*x*+3/2, *y*+1/2, −*z*+1/2; (v) *x*, *y*+1, *z*.
